# Fluorodeoxyglucose (FDG)-Avid Upper-Lobe Mass Mimicking Malignancy in an Elderly Patient With Allergic Bronchopulmonary Aspergillosis: A Case Report

**DOI:** 10.7759/cureus.101764

**Published:** 2026-01-18

**Authors:** Umut Ilhan, Şeyma Özden, Fatma Merve Tepetam, Bahar Aydogar, Erdogan Cetinkaya

**Affiliations:** 1 Pulmonology, University of Health Sciences, Yedikule Chest Diseases and Thoracic Surgery Training and Research Hospital, Istanbul, TUR; 2 Allergy and Immunology, University of Health Sciences, Süreyyapaşa Chest Diseases and Thoracic Surgery Training and Research Hospital, Istanbul, TUR

**Keywords:** allergic bronchopulmonary aspergillosis, aspergillus fumigatus sensitization, fdg avid pulmonary mass, mass like consolidation, pet ct imaging

## Abstract

Allergic bronchopulmonary aspergillosis (ABPA) is an immunologic lung disorder that typically occurs in patients with asthma or cystic fibrosis, yet atypical presentations may closely resemble pulmonary malignancy and result in extensive diagnostic evaluation.

We report the case of a 75-year-old male with chronic obstructive pulmonary disease (COPD) and long-term wood-dust and mold exposure who presented with fever, progressive cough, purulent sputum, and worsening dyspnea. Chest CT revealed a large heterogeneous mass-like consolidation in the right upper lobe, and PET-CT demonstrated intense fluorodeoxyglucose (FDG) uptake, raising a strong suspicion for lung cancer. Bronchoscopy and biopsy were non-diagnostic. Further evaluation showed peripheral eosinophilia, markedly elevated total IgE, and high *Aspergillus fumigatus*-specific IgE and IgG levels, establishing the diagnosis of ABPA. Treatment with oral corticosteroids and itraconazole led to striking clinical improvement and near-complete radiologic resolution of the mass.

This case highlights that ABPA may rarely present as a large FDG-avid pulmonary mass mimicking malignancy, and that considering ABPA in the differential diagnosis of tumor-like lesions may prevent unnecessary invasive procedures and delays in appropriate management.

## Introduction

Allergic bronchopulmonary aspergillosis (ABPA) is a hypersensitivity reaction to *Aspergillus* species (spp.), most commonly observed in patients with asthma and cystic fibrosis [1]. The disease is characterized by elevated IgE levels, eosinophilia, bronchiectasis, and recurrent pulmonary infiltrates [2]. Although ABPA predominantly affects patients with asthma, it may rarely occur in individuals without a prior history of asthma or atopic disease, particularly in those with chronic lung disease or environmental mold exposure. Although several imaging patterns of ABPA have been described, focal consolidations with mucus impaction and dilated pseudobronchi are uncommon and may resemble pulmonary malignancy [3]. This malignant-mimicking appearance may result from dense mucus plugging, marked inflammatory cell burden, and increased metabolic activity leading to fluorodeoxyglucose (FDG) avidity on PET imaging, which may lead to diagnostic uncertainty and extensive radiological and invasive evaluations.

We present an elderly patient with a focal FDG-avid pulmonary lesion initially suspected to be lung cancer who was ultimately diagnosed with ABPA following a comprehensive immunologic assessment.

## Case presentation

A 75-year-old male presented to the Emergency Department with a three-day history of high-grade fever (39.5°C), worsening cough, and marked fatigue. He reported progressive dyspnea, increased sputum production, and worsening cough for the past two months, limiting his daily activities. He denied weight loss but noted intermittent night sweats. His past medical history included hypertension and chronic obstructive pulmonary disease (COPD). There was no history of childhood or adult-onset asthma, allergic rhinitis, atopic dermatitis, or other atopic conditions. He had a 10-pack-year smoking history but had been abstinent for 50 years. The patient was a retired furniture worker with long-term exposure to wood dust and mold.

On physical examination, oxygen saturation was 90% on room air, and the respiratory rate was 18 breaths/min. Auscultation revealed decreased breath sounds in the right upper lung zone. Initial laboratory evaluation demonstrated eosinophils of 700 cells/µL, lymphocytes of 2600 cells/µL, and CRP of 18 mg/L. Liver and renal function tests were within normal limits (Table 1).

**Table 1:** Laboratory Findings at Presentation

Parameter	Result	Unit	Reference Range
Eosinophils	700	cells/µL	0-500
Lymphocytes	2600	cells/µL	1000-4000
C-reactive protein	18	mg/L	0-5
Total IgE	1494	IU/mL	<100
*Aspergillus fumigatus*-specific IgE	28.4	kU/L	<0.35
*Aspergillus fumigatus*-specific IgG	146	mg/L	<40
Liver function tests	Within normal limits	-	-
Renal function tests	Within normal limits	-	-

**Figure 1:**
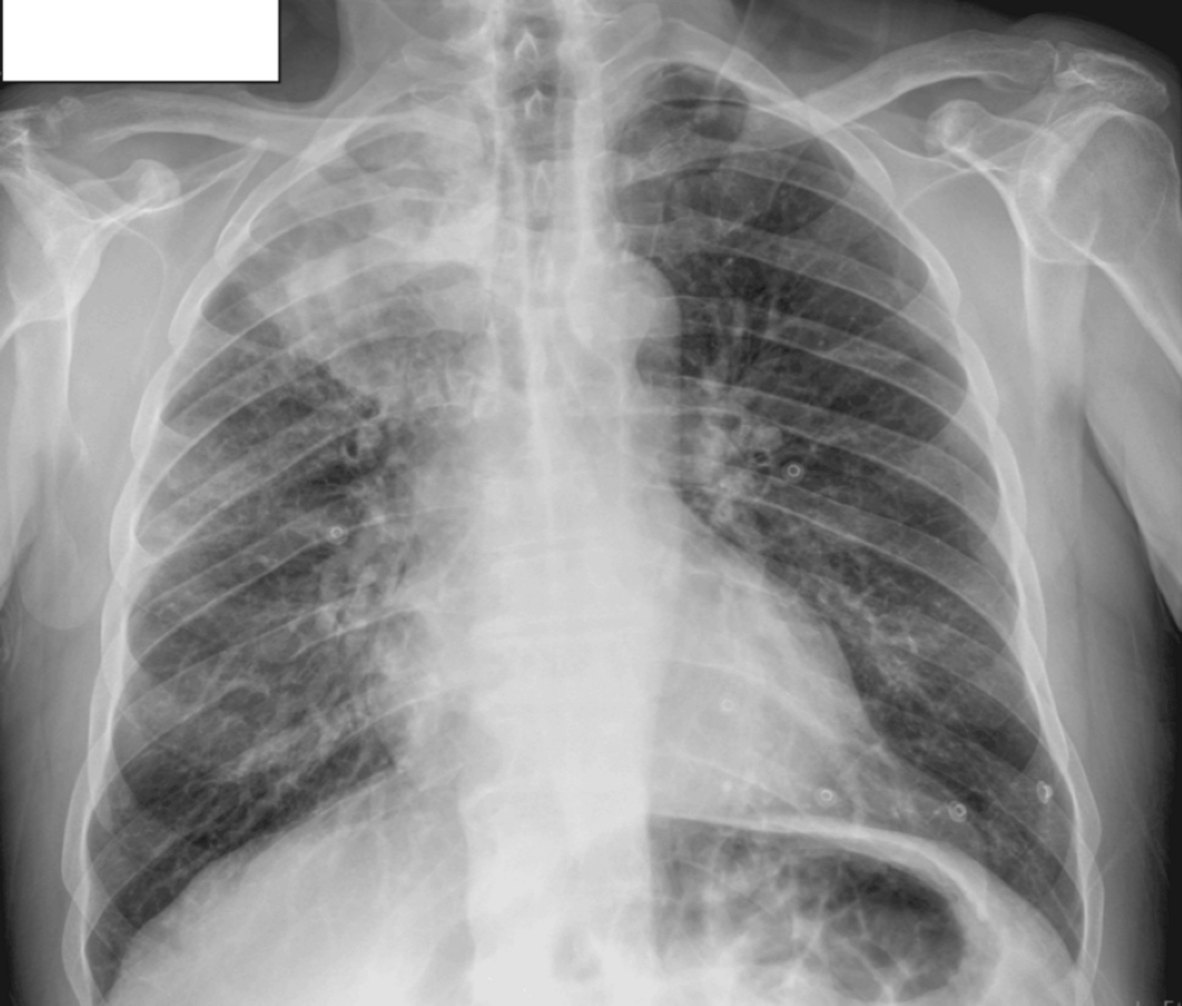
Initial Chest X-ray at Presentation

**Figure 2:**
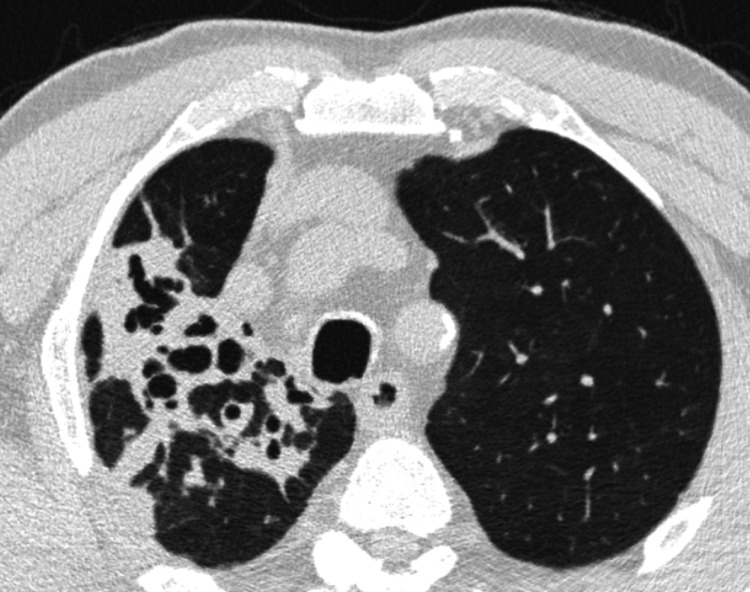
Initial Chest Computed Tomography at Presentation

A chest X-ray (Figure 1) showed consolidation in the right upper lobe, prompting a chest CT scan. CT imaging revealed a 10.5 cm focal consolidation with dilated pseudobronchi, accompanied by surrounding nodular opacities, fibro-atelectatic bands, and ground-glass densities (Figure 2). The presence of dilated pseudobronchi and surrounding inflammatory changes raised the possibility of mucus impaction-related disease in addition to malignancy. The patient was started on empirical antibiotic therapy for suspected lower respiratory tract infection and referred to the pulmonology clinic.

Following partial clinical improvement with CRP decreasing to 8 mg/L, bronchoscopy was performed due to persistent radiologic concern. Bronchoscopy revealed a patent anterior segment of the right upper lobe, while the apical and posterior segments appeared narrowed due to extrinsic compression. The apical segment was completely obstructed by a dense, inspissated mucus plug. Mechanical removal or mucolytic instillation was not attempted due to the distal location of the mucus plug and procedural safety considerations. Cytological examination and histopathological analysis of biopsy specimens were non-diagnostic. No fungal growth was observed in bronchial lavage cultures, and AFB smear and cultures were negative. Bronchoalveolar lavage galactomannan testing was not performed, as there were no clinical, radiologic, or laboratory findings suggestive of invasive aspergillosis.

**Figure 3:**
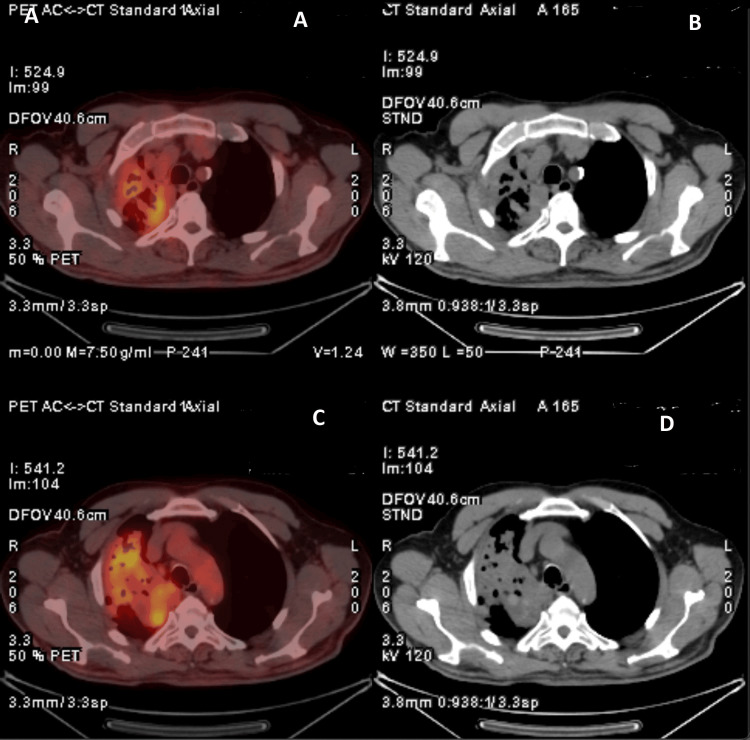
PET-CT Image Demonstrating Intense Fluorodeoxyglucose (FDG) Uptake (A, C) Axial 18F-FDG PET/CT fusion images demonstrating areas of increased metabolic activity within the thoracic lesion. (B, D) Corresponding axial non-contrast CT images at the same anatomical levels showing the structural correlates of the metabolically active regions. The combined assessment of PET and CT images enables simultaneous evaluation of metabolic and anatomical characteristics of the lesion.

PET-CT demonstrated increased FDG uptake within a focal pulmonary lesion occupying most of the right upper lobe, measuring 110 mm in diameter, with heterogeneous uptake (SUVmax 6.53) (Figure 3). FDG is a radiolabeled glucose analogue used in PET imaging to identify areas of increased metabolic activity; however, increased FDG uptake is also commonly observed in inflammatory and infectious pulmonary lesions and does not necessarily indicate malignancy.

Pulmonary function testing revealed preserved lung function with FEV₁ of 2920 mL (100% predicted), FVC of 3800 mL (97% predicted), and an FEV₁/FVC ratio of 76.

Given the patient’s thick brownish sputum, prolonged mold exposure, peripheral eosinophilia, markedly elevated total IgE (1494 IU/mL), and persistent radiologic infiltrates, ABPA was suspected. In the differential diagnosis, alternative considerations included primary lung malignancy, obstructive pneumonia secondary to mucus plugging, infectious pneumonias (particularly fungal and mycobacterial etiologies), and organizing pneumonia. Although skin prick testing for *Aspergillus fumigatus* was negative, *A. fumigatus*-specific IgE was markedly elevated at 28.4 kU/L and specific IgG at 146 mg/L (Table 1). A diagnosis of ABPA was established based on clinical, radiologic, and immunologic findings.

**Figure 4:**
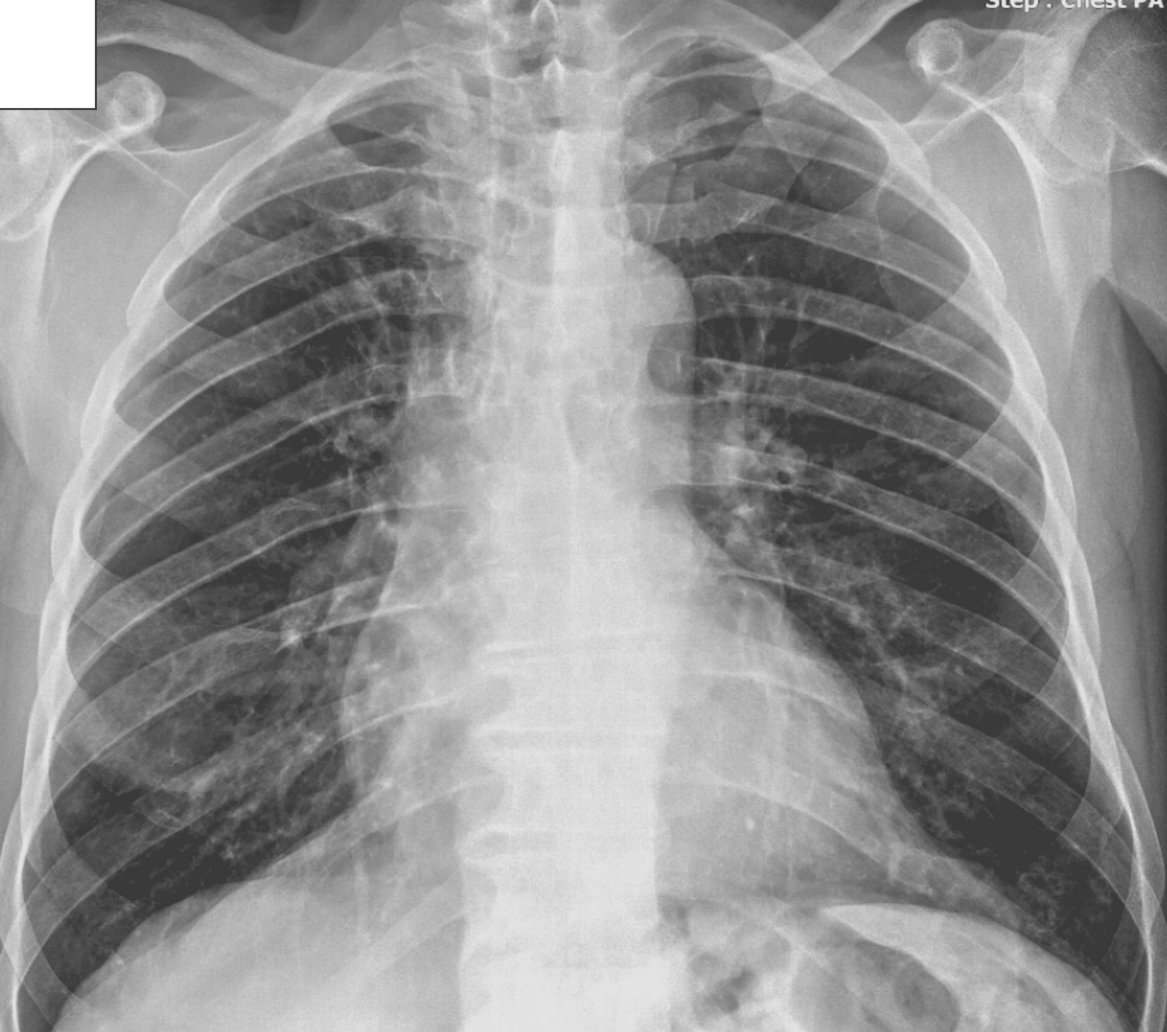
Follow-Up Chest X-Ray After ABPA Treatment ABPA, Allergic bronchopulmonary aspergillosis

**Figure 5:**
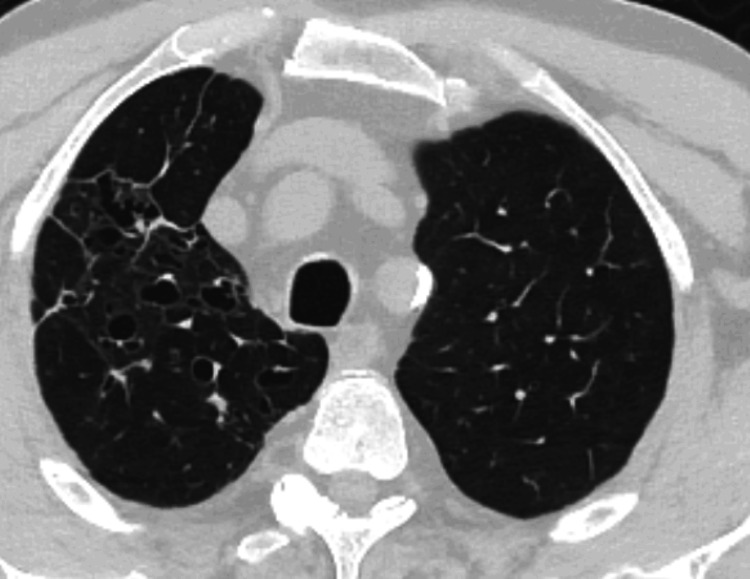
Follow-Up Chest Computed Tomography After ABPA Treatment ABPA, Allergic bronchopulmonary aspergillosis

The patient was treated with oral corticosteroids (0.5 mg/kg/day for two weeks, followed by gradual tapering to a maintenance dose of 4 mg/day) and itraconazole (200 mg three times daily for three days, followed by 200 mg twice daily) in accordance with established clinical practice guidelines for ABPA management [3]. The patient showed marked clinical improvement with resolution of cough and dyspnea. Follow-up chest radiographs (Figure 4) and CT imaging (Figure 5) demonstrated near-complete radiologic resolution of the focal consolidation. The residual linear bands and mild parenchymal contraction observed on follow-up imaging were interpreted as post-inflammatory fibrotic sequelae rather than active disease, supporting the diagnosis of a transient (fleeting) pulmonary infiltrate related to ABPA.

## Discussion

ABPA is an immunologic lung disease caused by a hypersensitivity reaction to *Aspergillus* spp., most commonly observed in patients with asthma or cystic fibrosis. However, ABPA has also been reported in individuals with COPD and even in those with structurally normal lungs, indicating that the condition extends beyond the traditionally recognized airway diseases [1,2]. The updated ISHAM-ABPA (International Society for Human and Animal Mycology-Allergic Bronchopulmonary Aspergillosis) Working Group 2024 guidelines emphasize that the clinical spectrum of ABPA is broader than previously understood and may include patterns that mimic neoplastic, infectious, or granulomatous disorders, particularly in older adults and in individuals with a history of significant mold exposure [3].

Radiologically, ABPA typically presents with central bronchiectasis, mucus impaction, and transient pulmonary infiltrates. High-attenuation mucus (HAM) remains a pathognomonic finding, although it is not universally present [3,4]. Increasingly, however, mass-like consolidations are being reported as atypical manifestations. The 2024 ISHAM guideline specifically describes “mass-like or tumor-mimicking opacities” as recognized but uncommon phenotypes that may lead to misdiagnosis as lung cancer and result in unnecessary invasive procedures [3].

In our patient, a large (10.5 cm), heterogeneous upper-lobe consolidation with surrounding nodular opacities and ground-glass densities was observed. The intense FDG uptake on PET-CT (SUVmax 6.53) further strengthened the suspicion of malignancy. Several studies have demonstrated that FDG avidity cannot reliably distinguish ABPA or fungal hypersensitivity reactions from malignant lesions, as inflammatory cell infiltration and fungal antigen-driven immune activation may generate high metabolic signals on PET imaging [5,6]. Consequently, PET-CT findings must be interpreted with caution in regions with endemic fungal exposure or in individuals with significant mold exposure, as emphasized in the ISHAM 2024 guidelines [3].

The diagnostic process was further complicated by a non-diagnostic bronchoscopy, a situation frequently encountered in ABPA cases presenting with mass-like consolidations. According to the ISHAM 2024 guideline, bronchoscopy may be non-contributory unless mucus impaction is sampled, and diagnosis should rely more heavily on immunologic criteria - such as elevated total IgE, peripheral eosinophilia, and patterns of *A. fumigatus*-specific IgE and IgG sensitization [3].

Our patient demonstrated markedly elevated *A. fumigatus*-specific IgE (28.4 kU/L) and IgG (146 mg/L), peripheral eosinophilia, increased total IgE, and persistent radiologic consolidation, fulfilling the diagnostic components outlined in the 2024 ISHAM criteria (Table 2). Notably, as highlighted in the revised guideline, a negative skin prick test does not exclude ABPA, since specific IgE assays remain more sensitive and reliable than skin testing in older individuals or those with chronic lung disease [3].

**Table 2:** Diagnostic Criteria for ABPA According to the ISHAM-ABPA Working Group International Society for Human and Animal Mycology-Allergic Bronchopulmonary Aspergillosis (ISHAM-ABPA) Working Group diagnostic criteria were applied for the diagnosis of allergic bronchopulmonary aspergillosis. Source: Adapted from [3] *A. fumigatus, Aspergillus fumigatus*; ABPA, Allergic bronchopulmonary aspergillosis

Section	Criteria/Description
Predisposing Conditions	Asthma, cystic fibrosis, COPD, bronchiectasis, or a compatible clinico-radiological presentation.
Essential Components	*A. fumigatus*-specific IgE ≥ 0.35 kUA/L; Serum total IgE ≥ 500 IU/mL
Other Components (Any Two)	Positive *A. fumigatus*-specific IgG; Blood eosinophil count ≥ 500 cells/µL (historical values acceptable); Thin-section CT consistent with ABPA (bronchiectasis, mucus plugging, high-attenuation mucus); Fleeting opacities on chest radiograph
Important Considerations	a) Expectoration of mucus plugs, finger-in-glove opacities, fleeting infiltrates, and lung collapse are strong indicators of ABPA. b) A positive type I skin test is acceptable when *Aspergillus*-specific IgE testing is unavailable. c) Serum total IgE < 500 IU/mL may still be acceptable if all other criteria are fulfilled. d) *A. fumigatus*-specific IgG can be detected using lateral flow assays or enzyme immunoassays. Cut-off values must be population-specific (e.g., ≥27 mgA/L for India, ≥60 mgA/L for Japan, ≥40 mgA/L for the UK). Manufacturer recommendations may be used when population-specific values are unavailable. e) High-attenuation mucus (HAM) is pathognomonic and confirms ABPA diagnosis even if other criteria are not met. Additional note: Elevated IgE against rAsp f1, f2, and f4 supports the diagnosis of ABPA and may be used as an additional diagnostic component.

Treatment with systemic corticosteroids and itraconazole resulted in dramatic radiological regression and symptomatic improvement, consistent with the therapeutic outcomes reported in previous ABPA cohorts [7]. The 2024 ISHAM guideline recommends the combination of oral corticosteroids and antifungal therapy in cases with extensive radiologic involvement or severe disease, as this approach has been associated with improved clinical responses and reduced exacerbations [3].

## Conclusions

This case underscores the importance of considering ABPA in the differential diagnosis of hypermetabolic pulmonary masses, especially in patients with COPD, environmental mold exposure, or eosinophilia. Failure to recognize atypical manifestations of ABPA can lead to prolonged diagnostic delay, unnecessary invasive interventions, and potentially inappropriate oncologic evaluations.
